# Drug Utilization and Drug Pricing in the Private Primary Healthcare System in Malaysia: An Employer Price Control Mechanism

**DOI:** 10.3389/fpubh.2020.551328

**Published:** 2020-12-07

**Authors:** Che Suraya Zin, Nor Hidayah Taufek, Siti Halimah Bux

**Affiliations:** Big Data Research Group, Department of Pharmacy Practice, Kulliyyah of Pharmacy, International Islamic University Malaysia, Kuantan, Malaysia

**Keywords:** drug utilization, uncontrolled drug pricing, employer insurance coverage, price control mechanism, private healthcare system in Malaysia

## Abstract

Uncontrolled drug pricing in the private healthcare system in Malaysia leads to high drug prices; however, its impact on employee drug utilization and employer reimbursement coverage is unclear. This study examined patterns of drug pricing and drug utilization among employees covered by employer medical insurance. A drug price control mechanism was also devised for the employer to ensure fair benefits to all parties without compromising the quality of patient care. This retrospective study was conducted among International Islamic University Malaysia (IIUM) community members who sought outpatient treatment at the IIUM panel of health clinics serviced by general practitioners from January 2016 to September 2019. Prescription data (drug type, dose, quantity, duration, price, and manufacturer), patient characteristics (age, sex, and diagnosis) and total charges were extracted from the claims database of PMCare, the insurance company managing IIUM medical claims. Patterns of commonly prescribed drugs, drug pricing, profit margins, and total charges per clinic visit were evaluated. Descriptive statistics were used, and all analyses were performed using Stata v15.1. There were a total of 161,146 prescriptions for 10,150 patients in the IIUM community during the study period (48.85% women, mean ± standard deviation; age: 26.33 ± 17.63 years). The most commonly prescribed drug was paracetamol (25.3%), followed by chlorpheniramine (9.46%), cetirizine (7.3%), diphenhydramine (6.13%), loratadine (4.57%), and diclofenac (4.36%). Generic paracetamol (500 mg), which serves as a prime example for details on drug pricing, is commonly charged between Ringgit Malaysia (RM) 5 and 10 for 10 tablets with a profit between 2,400 and 4,900% according to the average cost price of RM 0.20 per 10 tablets. Most patients were charged within the approved coverage limit of RM 45 per clinic visit, with only 2.41% of patients being charged with costs that exceeded this limit. Uncontrolled drug pricing in the private healthcare system in Malaysia indicates that drug prices differ greatly across private healthcare providers most of the prices were charged with high profit margins. Employers may consider a multilayer capping system to prevent inappropriate drug pricing, which will inevitably benefit patients clinically and economically and provide greater patient access to better drug treatment.

## Introduction

The healthcare system in Malaysia consists of both private and public sectors. The private sector is a non-subsidized sector that uses consumers' out-of-pocket money to pay for the services (1). This sector is also funded by non-profit institutions, private institutions, and private health insurance (2). The public sector is fully funded by the government, where patients receive their medicines either for free or at a nominal fee. In the private sector, it is solely free market forces that determine drug pricing, which results in markups or profit margins for drug prices to be higher in Malaysia than in other countries (3, 4). The lack of drug pricing regulations has resulted in healthcare professionals, wholesalers, and pharmaceutical companies in the private sector to set their own retail selling price (5, 6).

Private medical clinics in Malaysia usually have services provided by general practitioners that cater mostly to the self-paying public that uses fee-for-service methods of payment and private sector employees. Some private sector employees are medically covered by their employers through panel clinics or through insurance arrangements, which have, at times led to high drug charges primarily because payment is being made by a third party. This, however, depends on the insurance structure and its impact on drug prices.

The coverage for International Islamic University Malaysia (IIUM) employee medical benefits, which include hospitalization as well as both surgical and outpatient treatment by a general practitioner (GP), is arranged through an insurance company. For outpatients, there is a cap of RM 45 (equivalent to USD 10.58 [RM 1 = USD 0.23]) per GP clinic visit. However, IIUM employees have requested an increase in this coverage to RM 60 (USD 13.59), as the current amount approved is inadequate to cover the total charges (drug charges and consultation fee) for each outpatient clinic visit.

The present study aimed to evaluate the patterns of drug utilization and drug pricing among IIUM community members seeking treatment at IIUM panel clinics serviced by GPs, and to devise a price control mechanism for an employer sustainable reimbursement drug policy. The drugs most commonly used by the IIUM community, total charges per clinic visit, and profit margins for drug pricing were examined. In addition, the study investigated whether the current cap of RM 45 per clinic visit was adequate to cover employees' outpatient treatment.

## Materials and Methods

This study was approved by the IIUM Ethical Committee (IREC-2019-212). The results of the study are reported in an aggregated manner using de-identified data. Informed consent was not required as this study did not involve any direct patient interaction.

### Study Design and Data Source

This retrospective study was conducted in the IIUM community (employees and dependents) from January 2016 to September 2019, using the claims database from the insurance company PMCare, which manages and administers medical benefit programs to various clients. PMCare has about 650,000 members with a network of 2,000 affiliated medical providers in Malaysia (7).

IIUM is a client of PMCare and pays insurance premiums to PMCare to provide coverage for medical benefits for their employees. Given this arrangement, IIUM employees can seek treatment at any of the private medical clinics from the IIUM panel that are serviced by PMCare. They are not required to pay for the service rendered by the clinics if the amount is within the IIUM-approved coverage of up to RM 45 per clinic visit (USD 10.68), as this amount will be paid by PMCare.

The present study involved outpatient treatment that was provided by GPs. All IIUM employees (including dependents) who sought treatment from GPs during the study period were included. Data on prescriptions (drug type, dose, quantity, duration, frequency, price for each drug, and drug manufacturer), patient characteristics (age, sex, and diagnosis), and total charges (drug charges and consultation fees) were extracted from the PMCare database. The terms “drug” and “medication” were used interchangeably in this study. The term “patients” in this study refers to IIUM employees and their dependents.

### Outcome Measures

#### Number of Prescriptions, Patients, and Top Six Drugs Used by the IIUM Community

The total number of prescriptions and patients were assessed, and the most common drugs or medications prescribed by GPs for the IIUM community during the study period were identified. GP consultation fees for each clinic visit were also recorded.

#### Drug Pricing

Prices charged for each drug in the list of most commonly prescribed drugs were analyzed, and the manufacturer for each drug was identified. The price charged by the GPs was then compared with the cost price given by the manufacturer to evaluate the profit margin for each drug. Furthermore, the total cost incurred, using the price charged by the GP, for the top most commonly prescribed drug was calculated. According to these analyses, the framework for a drug pricing mechanism was proposed, and the expected cost saving using this mechanism was calculated.

#### Total Charges per Clinic Visit

The total charges (drug charges and consultation fees) per outpatient clinic visit for each patient were identified. The total charges per clinic visit per patient that were above the RM-45 cap were further examined to evaluate the type of drugs used and the number of drugs prescribed that led to the charges being higher than the approved amount.

### Data Analysis

Descriptive statistics were used to describe patient characteristics, whereby, the mean and standard deviation (SD) were used for continuous variables that were normally distributed and the median and interquartile range (IQR) were used for continuous variables that were non-normally distributed. Count and percentages were used to describe categorical variables. All statistical analyses were performed using Stata v15.1 (8).

## Results

### Number of Prescriptions and Patients

A total of 161,146 prescriptions for 10,150 patients (34% employees and 66% dependents) were identified during the study period from 2016 to 2019. There were 48.6% women (*n* = 4,958/10,150) and 51.2% men (*n* = 5,192/10,150) included in the study (Table 1). The mean ± SD age of the patients was 26.3 ± 17.6 years, with women (26.3 ± 16.8 years) being slightly older than men (25.9 ± 17.4 years).

**Table 1:** Patient demographics.

**Number of patients**	***n***	***%***
	**10,150**	**100**
**Gender**		
Male	5,192	51.15
Female	4,958	48.85
**Age (years old)**		
Mean	26.33	
Median	23	
Mode	4	
Range	1 to 78	
SD	17.1	
**Age group (years old)**		
0 to 9	2,002	20.65
10 to 19	2,272	23.44
20 to 29	1,401	14.45
30 to 39	1,504	15.51
40 to 49	1,375	14.18
50 to 59	898	9.26
≥60	242	2.50
**Most commonly drugs prescribed (≥4%)**		
Paracetamol	26,922	25.26
Chlorpheniramine	10,081	9.46
Cetrizine	7,784	7.3
Diphenhidramine	6,531	6.13
Loratadine	4,865	4.57
Diclofenac	4,646	4.36
**Most common diagnoses (≥2%)**		
Acute Upper Respiratory Infection, Unspecified	39,716	39.28
Dermatitis And Eczema	6,488	6.42
Infectious Gastroenteritis And Colitis	5,240	5.18
Acute Tonsillitis, Unspecified	3,457	3.42
Gastritis, Unspecified	3,046	3.01
Other And Unspecified Asthma	2,887	2.86
Essential (Primary) Hypertension	2,399	2.37
Other Specified Headache Syndromes	2,286	2.26
Unspecified Conjunctivitis	2,254	2.23

*Age among 9,694 patients, 456 patients with missing age*.

*Amoxicillin (antibiotics) has 4,661 prescriptions (4.37%). All other drugs were prescribed <4%*.

*All other diagnoses were <2%*.

The most common diagnoses reported by GPs among the IIUM community seeking treatment were acute upper respiratory infection (39%), dermatitis and eczema (6.42%), infectious gastroenteritis and colitis (5.18%), acute tonsillitis (3.42%), and gastritis (3.01%). The most frequent charges for the GP consultation fees per outpatient clinic visit were RM 20 (22.49%), followed by RM 15 (21.75%), RM 25 (16.30%), RM 30 (8.96%), RM 10 (8.92%), RM 35 (5.58%), and RM 18 (5.58%).

### Drugs Most Commonly Prescribed by GPs for the IIUM Community

**Figure 1:**
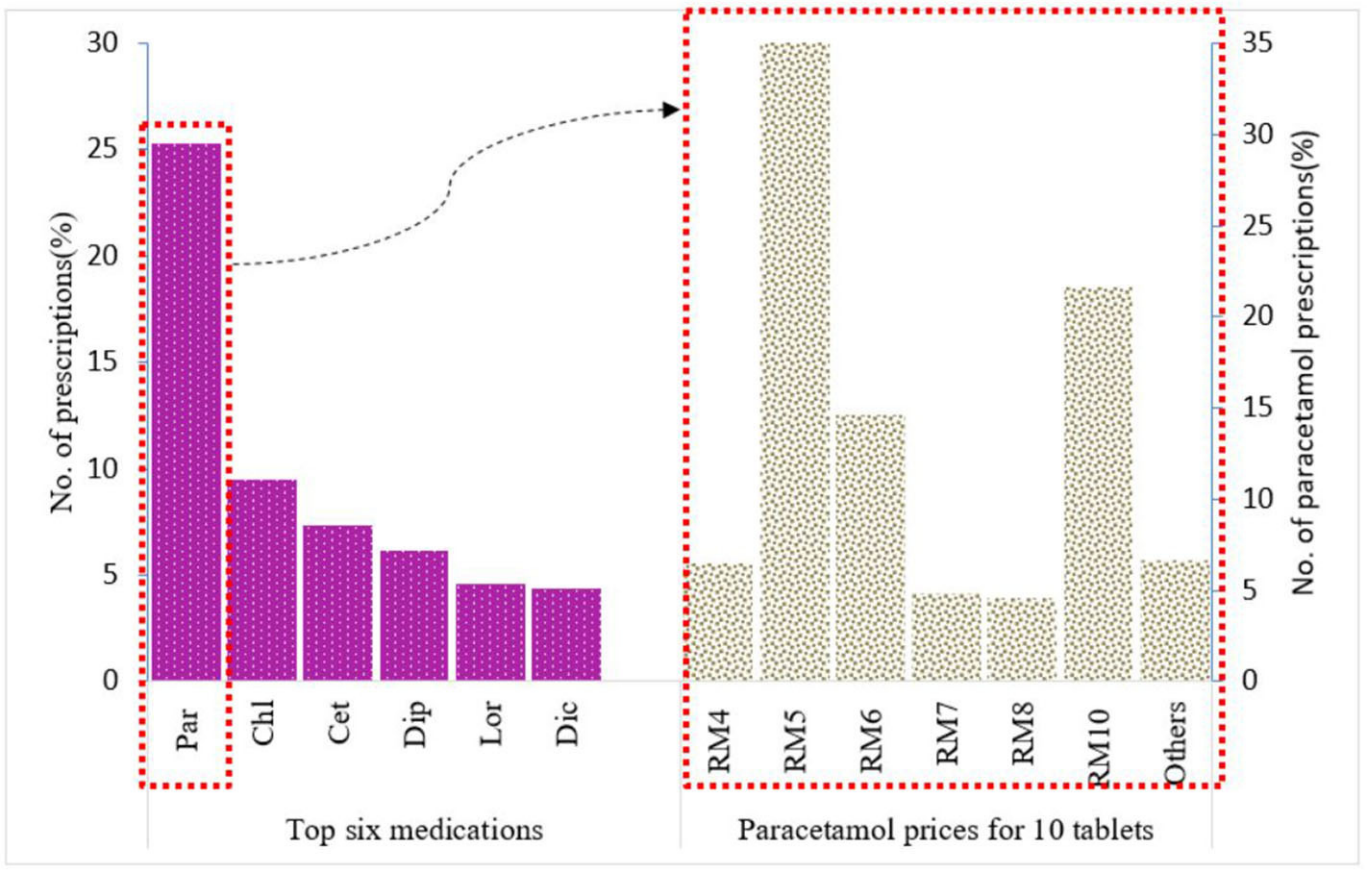
Top six medications prescribed for IIUM community and prices for paracetamol 500 mg 10 tablets from Duopharma Sdn Bhd.

There were ~1,500 drugs types included in the PMCare database during the study period. The drug most commonly prescribed by GPs for the IIUM community was paracetamol (25.3%), followed by chlorpheniramine (9.46%), cetrizine (7.3%), diphenhydramine (6.13%), loratadine (4.57%), and diclofenac (4.36%) (Figure 1). Most of these drug products were generic brands.

**Figure 2:**
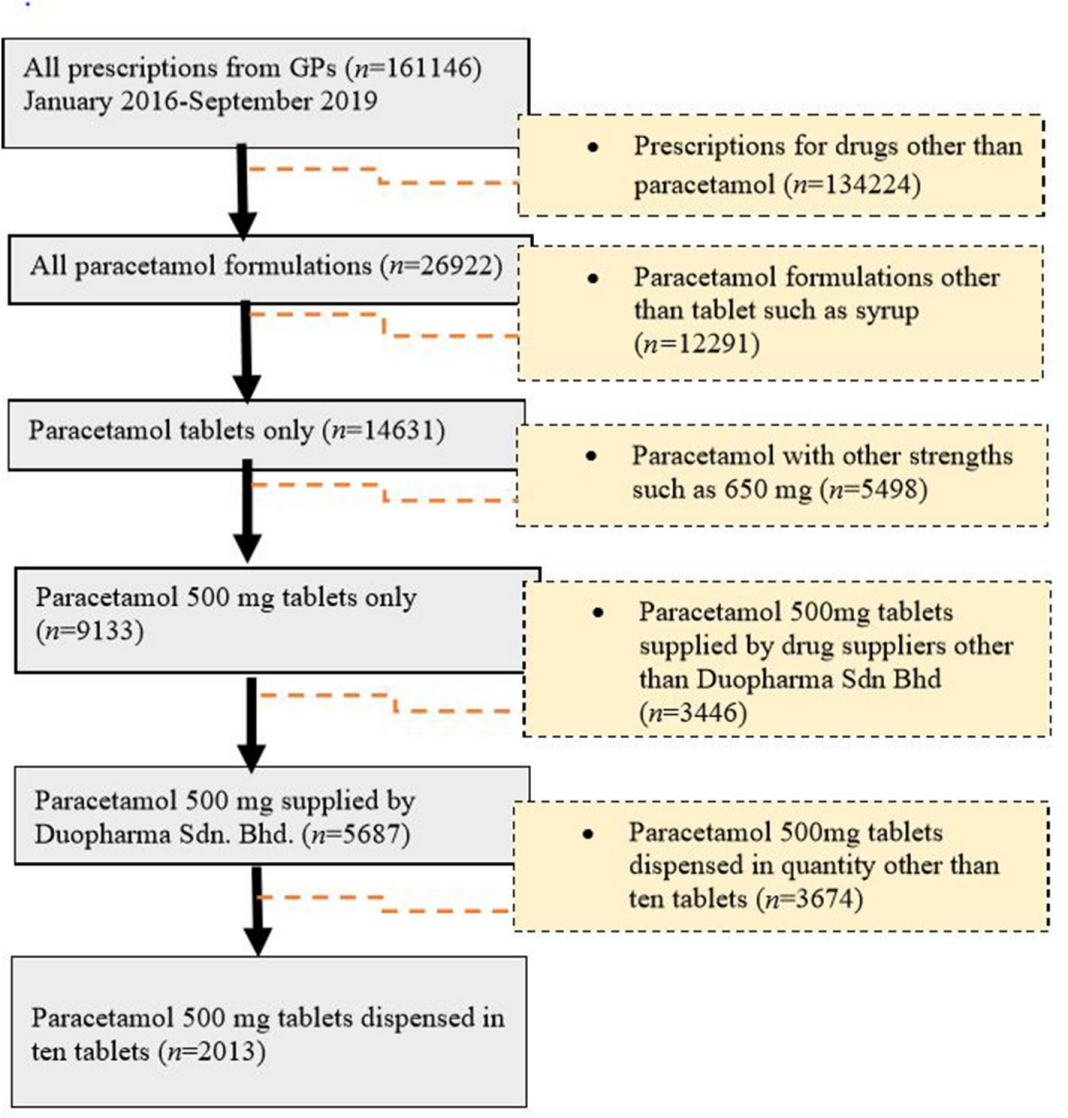
Cohort flow chart for paracetamol analysis.

Paracetamol was at the top of the list (*n* = 26,922, 25.26%) of the most commonly prescribed drugs. For paracetamol, there were different formulations with varying strengths, including 500-mg paracetamol tablets, 650-mg paracetamol tablets, 250-mg/5-mL paracetamol suspension, and a combination tablet containing 35-mg orphenadrine citrate and 450-mg paracetamol. Of these, the 500-mg paracetamol tablet (*n* = 9,133, 62.4%) was the most frequently prescribed (Figure 2). This product was further used as an example for the evaluation of drug pricing.

### Drug Pricing

Of the generic branded paracetamol 500 mg tablets prescribed, 62% (*n* = 5,687) were supplied by Duopharma Sdn Bhd. From this manufacturer, 35% (*n* = 2,013) of the paracetamol 500 mg tablets were dispensed as blister packs of 10 tablets. These blister packs were charged differently by the GPs, with prices ranging from RM 2 to RM 20. The most commonly charged price for this product was RM 5 (41.13%), followed by RM 10 (21.61%), RM 6 (14.65%), RM 4 (6.46%), RM 7 (4.87%), RM 8 (4.62%), and other prices (6.66%) (Figure 1).

The cost price for generic paracetamol 500 mg from Duopharma Sdn Bhd was RM 0.20 per 10 tablets (cost price at the time this study was conducted). If these 10 paracetamol tablets are charged at RM 5 (USD 1.19), it would result in a profit margin of ~2,400%, and if they are charged at RM 10 (USD 2.37), it would result in a profit margin of 4,900%, which is exceedingly high and does not adhere to the recommended selling price that ranges from RM 1 (USD 0.23) to RM 4 (USD 0.95) per blister pack of 10 tablets.

**Figure 3:**
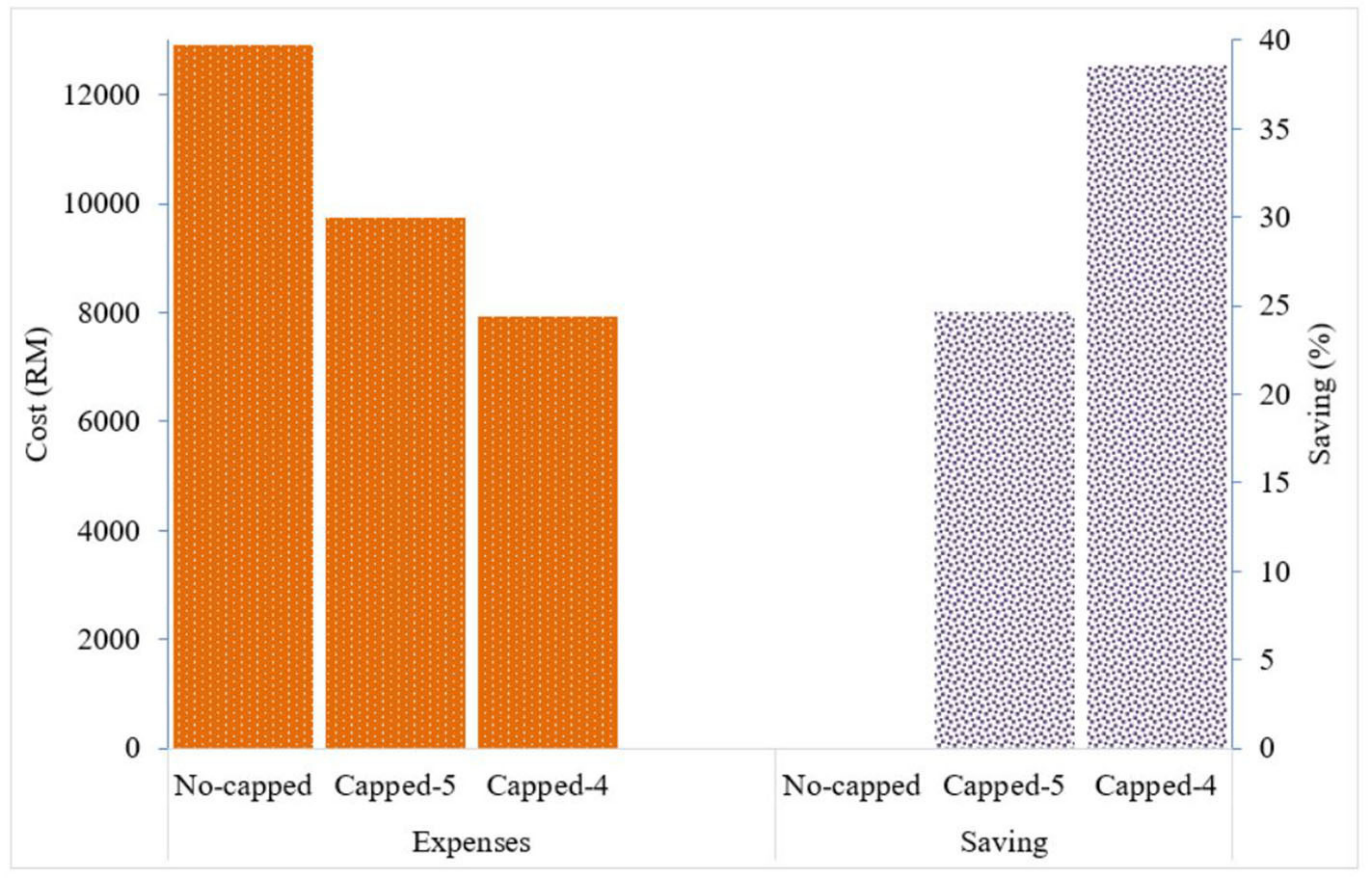
Drug expenses with the current pricing system and expected cost saving with the proposed capping system for generic paracetamol 500 mg 10 tablets.

Given the variation in the prices charged, the total expense for all paracetamol 500 mg packs of 10 tablets (*n* = 2,013) supplied by Duopharma Sdn Bhd during the study period was RM 12,926 (USD 3,067.76). If the price for this generic brand of paracetamol 500 mg is capped at RM 5 per 10 tablets, the total expense for this product would be RM 9,735 (USD 2,310.43), and if capped at RM 4, the total expense would be RM 7,941 (USD 1,884.66). This would result in an expected cost saving of ~24.7% with the proposed RM 5 cap, and an expected cost saving of ~38.6% with the proposed RM 4 cap (Figure 3).

Other commonly prescribed drug products, such as chlorpheniramine tablets, cetirizine tablets, diclofenac sodium tablets, diphenhydramine syrup, and loratadine tablets, were also mainly charged at RM 10 per 10 tablets or per bottle (for diphenhydramine syrup). The cost for these generic drugs ranged from RM 0.37 to RM 1.10 for 10 tablets, indicating a profit margin ranging from ~809 to 2,602%, which is similar to that of the paracetamol product. For diphenhydramine syrup, the cost is RM 6 per bottle, indicating a profit margin of 66.6% (Table 2).

**Table 2:** List of most common drugs, costs and profit margins.

**Name of drug**	**Manufacturer**	**Amount**	**Cost price**	**Selling price**	**Profit margin**
Chlorpheniramine tablet	Sunward	10 s	0.37	RM 10	2,602%
Diphenhydramine syrup	Xepa- Soul Pattinson	120 ml	6.00/bottle	RM 10	66.6%
Diclofenac sodium 50 mg tablet	YSP	10 s	0.97	RM 10	930%
Loratadine tablet	Pharmaniaga	10 s	0.90	RM 10	1,011%
Cetirizine tablet	Xepa-Soul	10 s	1.10	RM 10	809%

*The cost price was from the manufacturer supplying a particular drug*.

*The selling price was based on the most common selling price for a particular drug*.

*10s, 10 tablets; RM, Ringgit Malaysia*.

### Total Charges for Outpatient Clinic Visits

**Figure 4:**
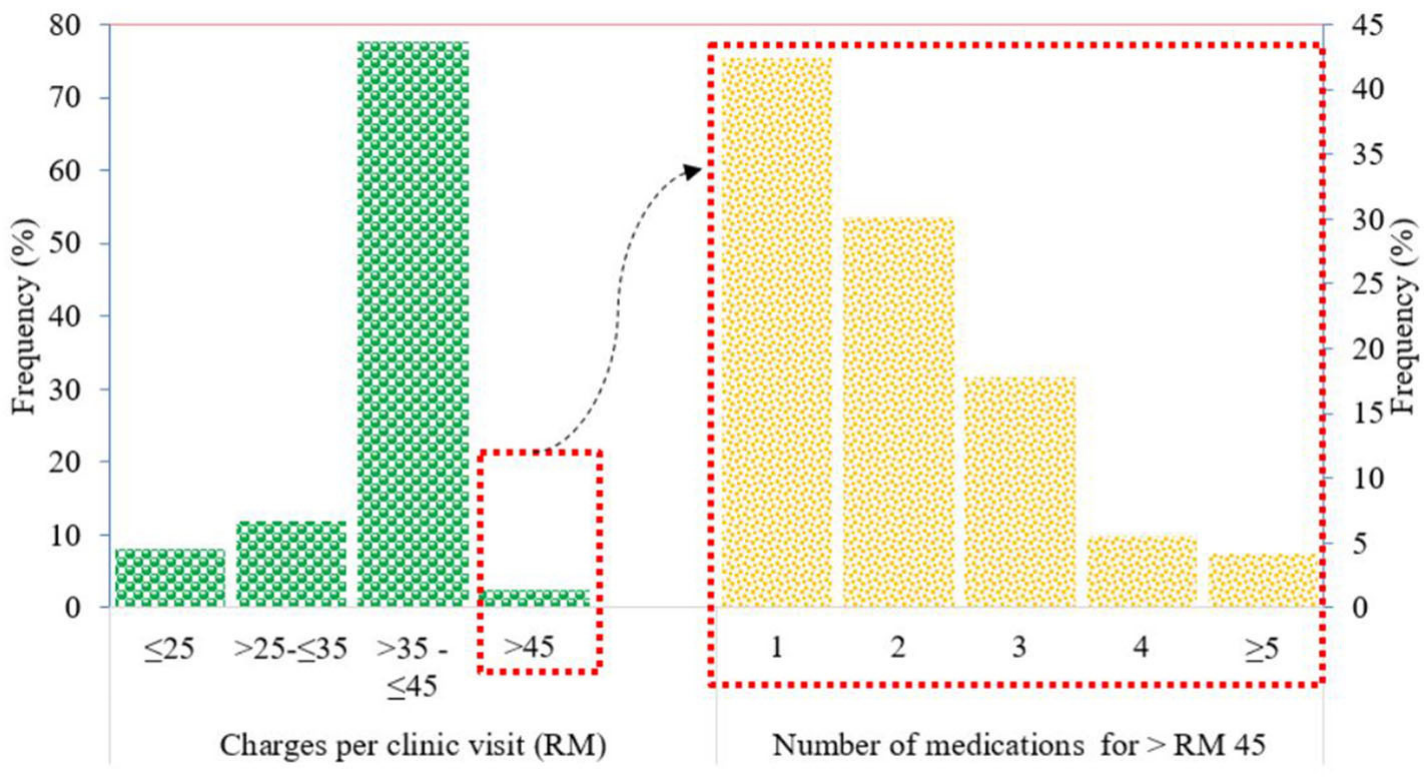
Total charges per clinic visit and number of drugs prescribed for total charges >RM 45.

The amount charged by the GPs for treating members of the IIUM community ranged from RM 25 to more than RM 100 per clinic visit. The most common range of amounts charged per clinic visit was >RM 35 to ≤ RM 45 (77.6%), followed by >RM 25 to ≤ RM 35 (11.9%), ≤ RM 25 (8%), and >RM 45 (2.41%) (Figure 4).

Patients with charges exceeding the IIUM-approved coverage of RM 45 per clinic visit were mainly prescribed branded drugs such as gliclazide (Diamicron®) for diabetes and irbesartan (CoAprovel®) for hypertension. Most of these patients were only prescribed one (42.47%) of these branded drugs for a short duration of treatment, and this exceeded the approved coverage amount of RM 45 per clinic visit.

Patients prescribed with two drugs, where their total charges exceeded the approved coverage of RM 45 per clinic visit, were usually prescribed with commonly used drugs such as generic paracetamol and cetirizine for minor cough and cold conditions. The amount exceeding RM 45 was because of the charge of ≥RM 10 for each drug, making the drug charge approximately RM 20 for the two products. Along with consultation fees, this charge would exceed RM 45 per clinic visit.

For patients prescribed with three or more drugs, where the total charges exceeded RM 45 per clinic visit, it was observed that antibiotics such as cephalexin and erythromycin were often prescribed along with the commonly used drugs such as paracetamol and cetirizine. The prices for antibiotics ranged from RM 10 to RM 30 for a 5- to 7-day course; thus, the total charge per clinic visit would exceed RM 45.

## Discussion

This study showed that uncontrolled drug pricing in the private healthcare system in Malaysia has resulted in varying ranges of drug prices among GPs at private medical clinics. The high profit not only costs a lot to the employers but also to the patients who have to pay out-of-pocket for the drugs. This suggests that mechanisms should be developed to ensure accessibility and affordability and to provide fair benefits to all parties without compromising the quality of patient care (9, 10).

There are several price control mechanisms commonly used to decrease drug cost expenditure by the government, including reference pricing (internal and external), tiered formularies, and price caps (11–13). Internal reference pricing uses price comparisons across equivalent drugs within the same country, and external reference pricing uses price comparisons of similar drugs in one country with those in another country or in a group of countries (14). If higher-cost drugs are prescribed, patients pay the difference. For tiered formularies, patients face incrementally higher copayments for different treatment options, for example, low copayments with first-tier drugs within a class and higher copayments for second- and third-tier drugs within a class (15). With regard to price caps, some countries set price caps on generic drugs and/or regulate the maximum reimbursement rate to limit pharmaceutical companies from charging high prices (16–18). Malaysia is just beginning the planning of its drug price control mechanism, which still requires additional time and stages before it can be implemented. The findings from this study can form the preliminary groundwork for developing the price control mechanism.

Using paracetamol as a prime example in evaluating drug pricing details, the present study showed that capping the price for this generic drug at RM 4 or at RM 5 per 10 tablets would result in cost savings of ~25 to 40% for the employer. If the current expenditure for drug reimbursement is ~1 million per year, using this example, IIUM could save between RM 250,000 and RM 400,000 per year. The capping system could also be extended to the price of other commonly used drugs for an even greater cost savings. The recommended capping of prices would still allow GPs to have profit margins between 1,900 and 2,400%. Therefore, all parties (patients, employers, and GPs) would still benefit from the system, which would be a win-win outcome. While these commonly used drugs are mainly used for acute minor illnesses, the proposed capping system will not include drugs prescribed for chronic illnesses, and this may limit the treatment options with the proposed system (11).

For chronic illnesses, prescribing of low-cost generic drugs that have similar therapeutic activity should be promoted. These products have similar efficacy profiles to originators or branded drugs; thus, patient care will not be undermined, and substantial saving can be achieved (19–21), as well as improved prescribing efficiency (22, 23). Several initiatives have been introduced to enhance the generic prescribing in Western European Countries, such as demand-side measures (24) which include educational activities, prescribing targets, prescribing restrictions, compulsory International Nonproprietary Name (INN) prescribing and financial incentives for patient co-payment differentials (20, 21). Malaysia should learn from these measures and choose the most effective measure to manage its pharmaceutical cost.

The needs for prescribing of low-cost generic drugs was also shown in the current study in which, with only one expensive branded drug, total charges have already exceeded the approved coverage of RM 45 per clinic visit. If low-cost generic drugs were prescribed, the approved amount would allow patients to receive more than one drug for their illnesses. Information about generic and branded drugs need to be conveyed to patients. If they prefer the branded drug, then they pay the difference. Concerning the small percentage of patients (2.41%) exceeded RM 45 per clinic visit shown in the present study, it may be because of the unrecorded out-of-pocket payments made by the patients. This may underestimate the real number of patients exceeding the coverage amount. Thus, GPs are required to report the exact details of what was done in practice.

Patients may benefit from the proposed capping system with regard to the number of medications they can access per clinic visit. Prior to capping, they may receive only two types of medications with the approved coverage of RM 45 per visit (~RM 20 for two medications and the balance for consultation fees). With the capping system, they may be able to receive three to four types of medications per visit (RM 5 for each medication for a total charge for three to four drugs of ~RM 20 and the balance for consultation fees). Although the consultation fees may vary depending on the complexity of cases, the current study showed that RM 20 (range: RM 10 to RM 35) was the most commonly charged consultation fee. This can accommodate the coverage amount of RM 45 per clinic visit for drug charges as well as consultation fees.

**Figure 5:**
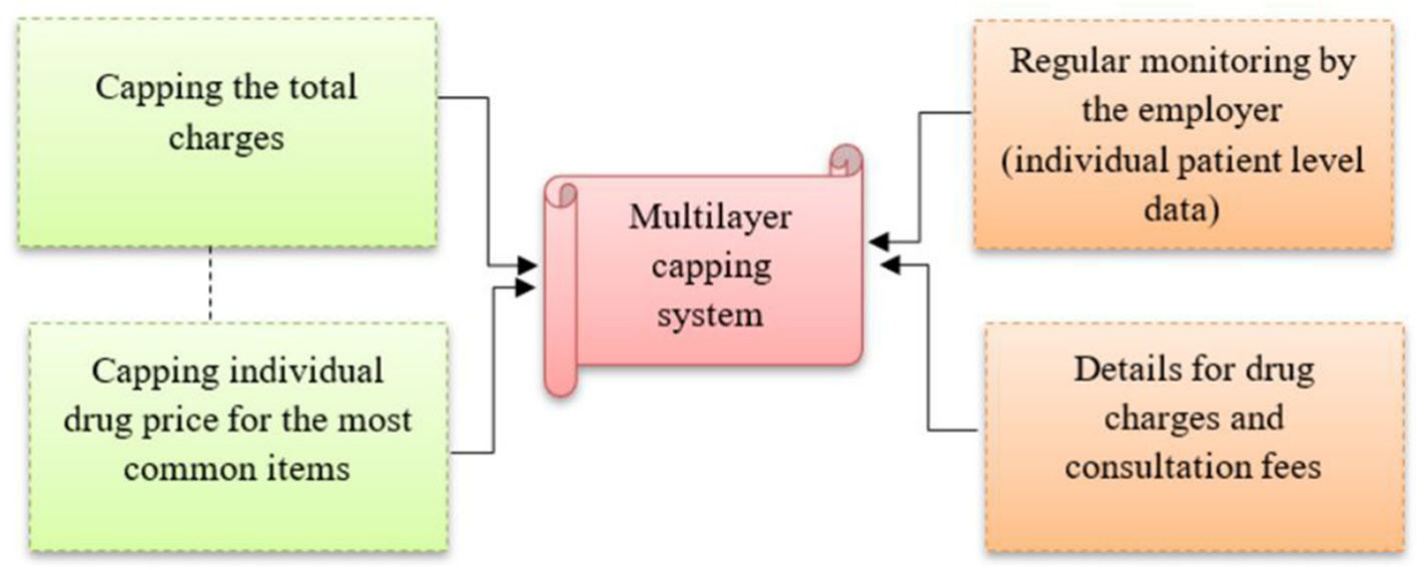
Framework for multilayer capping system.

The proposed capping of individual drug pricing in this study will be in addition to capping the total approved amount covered of up to RM 45 per clinic visit (Figure 5). This multilayer capping system will prevent inappropriate drug pricing, and improve patient surplus and social welfare (25). Without any control on drug pricing, the current approved coverage (RM 45 per visit) or the revised coverage (RM 60 per visit as requested by employees) will not make a significant difference to the current situation, as the price of drugs and consultation fees can be manipulated to the maximum allowable coverage during every clinic visit.

Other measures are also required to facilitate the multilayer capping system, including regular monitoring of the pattern of drug use by the employees. Data on patient claims at the individual level need to be obtained from the insurance company for monitoring purposes. Also, GPs are required to provide details on individual drug charges and consultation fees. These detailed charges have already been in practice at private tertiary hospital settings in Malaysia, but they are still lacking in private primary care settings. Employers need to emphasize this before renewing the appointment of panel clinics. At the national level, efforts to contain drug pricing in the private sector may be conducted using several different methods including a database with transparent drug pricing that requires disclosure of the recommended retail price by the manufacturer or wholesaler, and is made available for public access (26). In addition, public announcements and emphasis on drug price transparency can also provide options that are both effective and efficient for consumers in their procurement, and which in the long term would lead to healthy competition that may decrease medicine prices and increase consumer access to affordable medicines (27).

Apart from the above measure of price capping and prescribing of low-cost generic drugs in an effort to contain the pharmaceutical cost, the employer or the payer may also consider the drug formulary that lists of all essential medicines/well accepted drugs for private primary care setting. While this list is available within the public health system in Malaysia, it is lacking within the private health sector. The list provides high quality evidence based recommendations to prescriber which is in line with the recommendation of the Lancet's Commission on Essential Medicines policies to achieve Sustainable Development Goal 3.8 on universal access to safe, effective, quality and affordable essential medicines and vaccines (28). Concerning the private primary care setting, the range of medicines can be smaller than the range in tertiary care settings, because primary care typically deals with simple clinical cases and complicated cases are referred to tertiary settings. Nevertheless, the “Wise List” of essential medicines in Sweden is applicable for both primary and specialized care in Stockholm Healthcare Region and this list has gained strong acceptance and high adherence among prescribers (29).

This study provides important information on the patterns of drug use and drug pricing among private medical clinics, which can serve as paramount evidence in strategizing the price control and reimbursement drug policy. For the private primary healthcare system in Malaysia, this provides the groundwork for them to move forward in tackling the issue of high drug cost and uncontrolled drug pricing in the private sector. A number of limitations to this study need to be acknowledged, including the database accessed for this study that may have only captured the total charges within the maximum allowable coverage (≤ RM 45) for reimbursement from the insurance company, thus underestimating the actual number of patients who may have exceeded the maximum coverage amount. There is also a possibility that drugs prescribed within the exceeded coverage were not captured in the database. However, data in this study from a small number of patients who had exceeded the maximum coverage amount provide some basic information on patterns of drug use and drug pricing among this group, making the results of study beneficial in providing information for the future. Further, this study included data primarily from private GPs, which limits its generalization to other healthcare providers or settings such as private community pharmacies.

## Conclusion

Uncontrolled drug pricing in the private healthcare system in Malaysia indicates that drug prices differ greatly across healthcare providers at private medical clinics, primarily those with high profit margins. The multilayer capping system covering total claim amounts and selected individual drug charges will prevent inappropriate drug pricing in the private sector, and this will inevitably benefit patients clinically and economically, as patient accessibility to better drug treatment will become possible. Prescribing of generic drugs should be promoted and a drug formulary for primary care setting should also be considered to further improve cost containment and prescribing efficiency. The current maximum allowable coverage for the IIUM community at RM 45 per clinic visit may need to be revised when further evidence is available for justification. IIUM can be the first in implementing its own drug price control mechanism at the institutional level to ensure rational drug use among its employees, and at the same time, ensure financially sustainable reimbursement drug policy for IIUM. This could become a blueprint for other employers as well as lay the groundwork for the country in setting a national drug price control mechanism.

## Data Availability Statement

The datasets presented in this article are not readily available because request to access the datasets should be directed to International Islamic University Malaysia Research Ethical Committee. The de-identified data could be shared with interested researchers after obtaining the approval from the above ethical committee. The reason for the restriction on public data deposition is due to the privacy and confidentiality of patients' health data. Requests to access the datasets should be directed to http://www.iium.edu.my/centre/irec.

## Ethics Statement

The studies involving human participants were reviewed and approved by International Islamic University Malaysia Research Ethics Committee. Written informed consent from the participants or participants' legal guardian/next of kin was not required as this study did not involve any direct patient interaction.

## Author Contributions

CZ initiated and developed the research questions and study design, conducted data management and analysis, and led on drafting the manuscript. NT and SB contributed to the data acquisition, interpretation of the data, critically revised the manuscript and approved the final version submitted for publication. All authors contributed to the article and approved the submitted version.

## Conflict of Interest

The authors declare that the research was conducted in the absence of any commercial or financial relationships that could be construed as a potential conflict of interest.

## References

[1] Hassali MA Tan CS Wong ZY Saleem F Alrasheedy AA Pharmaceutical pricing in Malaysia. In: Babar Z-U-D, editor. Pharmaceutical Prices in the 21st Century. Cham: Springer International Publishing (2015), 171–88.; 10.1007/978-3-319-12169-7_10

[2] Jaafar S Mohd Noh K Muttalib AK Healy ONJ Malaysia health system review. Health Syst Trans. (2013) 3:1.

[3] Bahri S Use of Health Economics Data in Listing and Pricing of Pharmaceuticals: Ministry of Health Perspective. Monash Health Economic Forum 2013. Monash University Malaysia, Bandar Sunway (2013).

[4] Babar ZU Ibrahim MI Singh H Bukahri NI Creese A. Evaluating drug prices, availability, affordability, and price components: implications for access to drugs in Malaysia. PLoS Med. (2007) 4:e82. 10.1371/journal.pmed.0040082 17388660 PMC1831730

[5] Hassali MA Shafie AA Babar ZUD Khan TM A study comparing the retail drug prices between Northern Malaysia and Australia. J Pharm Health Serv Res. (2012) 3:103–7. 10.1111/j.1759-8893.2011.00080.x

[6] Kolassa E Prices politic, and problems-a pricing philosophy. J Pharm Mark Pract. (1997) 1:21–7. 10.1300/J289V01N01_03

[7] PMCare Available online at: https://www.pmcare.com.my/about (accessed March 31, 2020).

[8] StataCorp Stata: Release 15. College Station, TX: StataCorp LLC (2015).

[9] Prada SI Soto VE Andia TS Vaca CP Morales ÁA Márquez SR. Higher pharmaceutical public expenditure after direct price control: improved access or induced demand? The Colombian Case. Cost Effect Resour Alloc. (2018) 16:1–8. 10.1186/s12962-018-0092-0 29507533 PMC5833155

[10] Wiedenmayer K Access to Medicines Medicine Supply: Lessons Learnt in Tanzania and Mozambique. Switzerland (2004). Available online at: http://www.tzonline.org/pdf/accesstomedicinesmedicinesupply.pdf (accessed March 31, 2020).

[11] Luiza V Chaves LA Silva RM Emmerick ICM Chaves SC De Araújo F. Pharmaceutical policies : effects of cap and co-payment on rational use of medicines (Review) summary of findings for the main comparison. Cochrane Datab Syst Rev. (2015) 2015:CD007017. 10.1002/14651858.CD007017.pub2 25966337 PMC7386822

[12] Danzon PM. Differential pricing of pharmaceuticals: theory, evidence and emerging issues. PharmacoEconomics. (2018) 36:1395–405. 10.1007/s40273-018-0696-4 30062518

[13] Sabine V Fair prices for medicines? Exploring competent authorities' and public payers' preferences on pharmaceutical policies. Empirica. (2019) 46:443–69. 10.1007/s10663-019-09446-5

[14] David B Jullien B Lozachmeur JM. Health insurance and diversity of treatment. J Health Econ. (2016) 47:50–63. 10.1016/j.jhealeco.2016.01.003 26930001

[15] Morgan S Hanley G Greyson D. Comparison of tiered formularies and reference pricing policies: a systematic review. Open Med. (2009) 3:131–39. 21603047 PMC3090119

[16] Brekke KR Grasdal AL Holmas TH Regulation and pricing of phar- maceuticals: reference pricing or price cap regulation? Eur Econ Rev. (2009) 53:170–85. 10.1016/j.euroecorev.2008.03.004

[17] Brekke KR Königbauer I Straume OR. Reference pricing of pharmaceuticals. J Health Econ. (2007) 26:613–42. 10.1016/j.jhealeco.2006.11.003 17188769

[18] Hu J Mossialos E. Pharmaceutical pricing and reimbursement in China: when the whole is less than the sum of its parts. Health Policy. (2016) 120:519–34. 10.1016/j.healthpol.2016.03.014 27080345

[19] Bennie M Godman B Bishop I Campbell S. Multiple initiatives continue to enhance the prescribing efficiency for the proton pump inhibitors and statins in Scotland. Exp Rev Pharm Outc Res. (2012) 12:1. 10.1586/erp.11.98 22280202

[20] Brian G Payers endorse generics to enhance prescribing efficiency: impact and future implications, a case history approach. Gen Biosim Init J. (2012) 1:69–83. 10.5639/gabij.2012.0102.017

[21] Godman B Campbell S Suh HS Finlayson A Bennie M Ongoing measures to enhance prescribing efficiency across Europe: implications for other countries. J Health Technol Assess. (2013) 2013:27–42.

[22] Leporowski A Godman B Kurdi A MacBride-Stewart S Ryan M Hurding S. Ongoing activities to optimize the quality and efficiency of lipid-lowering agents in the Scottish National Health Service: influence and implications. Exp Rev Pharm Outc Res. (2018) 18:655–66. 10.1080/14737167.2018.1501558 30014725

[23] Godman B Kurdi A McCabe H Johnson CF Barbui C MacBride-Stewart S. Ongoing initiatives within the scottish national health service to affect the prescribing of selective serotonin reuptake inhibitors and their influence. J Comp Effect Res. (2019) 8:535–47. 10.2217/cer-2018-0132 31023070

[24] Godman B Wettermark B van Woerkom M Fraeyman J Alvarez-Madrazo S Berg C. Multiple policies to enhance prescribing efficiency for established medicines in europe with a particular focus on demand-side measures : findings and future implications. Front Pharm. (2014) 5:106. 10.3389/fphar.2014.00106 24987370 PMC4060455

[25] Xu C Yang H Wang X Effects of price cap regulation on the pharmaceutical supply chain. J Busin Res. (2019) 97:281–90. 10.1016/j.jbusres.2018.01.030

[26] Pharmaceutical Services Division Malaysian National Medicine Policy 2012. (2012). Available online at: https://www.pharmacy.gov.my/v2/sites/default/files/document-upload/buku-dunas.pdf (accessed March 31, 2020).

[27] Hinsch M Kaddar M Schmitt S. Enhancing medicine price transparency through price information mechanisms. Global Health. (2014) 10:34. 10.1186/1744-8603-10-34 24885767 PMC4019947

[28] Veronika WJ Hogerzeil HV Gray AL Bigdeli M Ewen MA de Joncheere CP Essential medicines foruniversal health coverage. Lancet. (2017) 389:3–76. 10.1016/S0140-6736(16)31599-9 27832874 PMC7159295

[29] Eriksen J Gustafsson LL Ateva K Bastholm-Rahmner P Ovesjö ML Jirlow M. High adherence to the'wise list'treatment recommendations instockholm: a 15-year retrospectivereview of a multifaceted approachpromoting rational use of medicines. BMJ Open. (2017) 7:e014345. 10.1136/bmjopen-2016-014345 28465306 PMC5775463

